# Royal Waterloo Hospital for Children and Women

**Published:** 1904-06-25

**Authors:** 


### ROYAL WATERLOO HOSPITAL FOR CHILDREN AND WOMEN.

A festival dinner in aid of this hospital took place on Monday at the Savoy Hotel. The Duke of Argyll presided, and a distinguished company was present. The Chairman, in proposing "Prosperity to the Hospital," said that they knew the ever-increasing difficulty of meeting the demands of the population of London. Other countries had seen fit to put under municipalities and communes all such institutions as that, but we kept to the old plan almost entirely of supporting hospitals by voluntary contributions. One of the objections to our system was that we might make it too easy for the recipients of bounty to have everything done for them, instead of doing it for themselves. The Waterloo Hospital did what it could, at all events, to prevent sick children from growing up into sick men and women. That was an argument which, he thought, countervailed any others in regard to any little social danger connected with helping people too much. It was very important that we should make the rising generation as well and strong as possible. They wanted a hospital with 200 beds, where every case could be treated and carefully tended and examined. Much had been done already, and only about half the building remained to be put up. An inspection of the hospital would show what an immense amount of good was being done in it. Mr. J. Topham-Richardson, treasurer, said, in reply to the toast, that they wanted £50,000, but their first outlay would be one of £30,000, towards which they already had about half subscribed. Sir E. Durning-Lawrence, M.P., chairman of the hospital, also replied, and made an earnest appeal for support for the charity, and other toasts followed. During the evening subscriptions and donations amounting to over £1,400 were announced.

